# Case Report: A giant myopericytoma involving the occipital region of the scalp - a rare entity

**DOI:** 10.12688/f1000research.10505.1

**Published:** 2016-12-22

**Authors:** Sunil Munakomi, Pramod Chaudhary

**Affiliations:** 1 Department of Neurosurgery, College of Medical Sciences, Chitwan, Nepal

**Keywords:** myopericytoma, subcutaneous lesion, scalp

## Abstract

Herein we report a rare case of a giant myopericytoma presenting in a 16-year-old girl as a slowly progressive swelling involving the scalp in the occipital region. It was managed by complete excision. Histological examination of the lesion revealed  spindle-shaped cells forming characteristic rosettes around the blood vessels, and positive staining with smooth muscle actin.

## Introduction

Myopericytoma is a rare entity. It mostly involves the skin and subcutaneous tissue of the distal extremities, torso, head and neck regions ^1– 3^. Rarely does it involve the visceral sites ^4, 5^. The spindle shaped cells of a myopericytoma show characteristic perivascular rosettes ^6, 7^. Though mostly benign, rare cases of its malignant counterpart have been described ^8^. We report a case of a giant myopericytoma involving the occipital region of the scalp of a young female, with good post-operative outcome following its complete excision. We believe this is the first case report of a giant myopericytoma involving this region.

## Case report 

**Figure 1.**
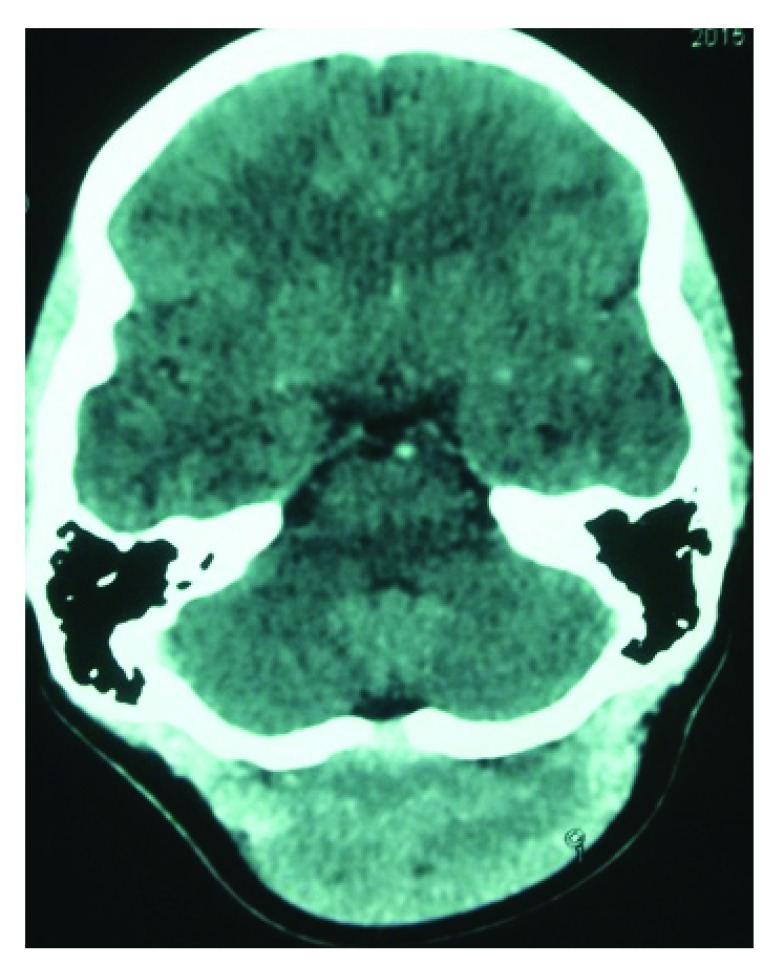
CT image showing homogeneously enhanced lesion in the scalp of occipital region.

**Figure 2.**
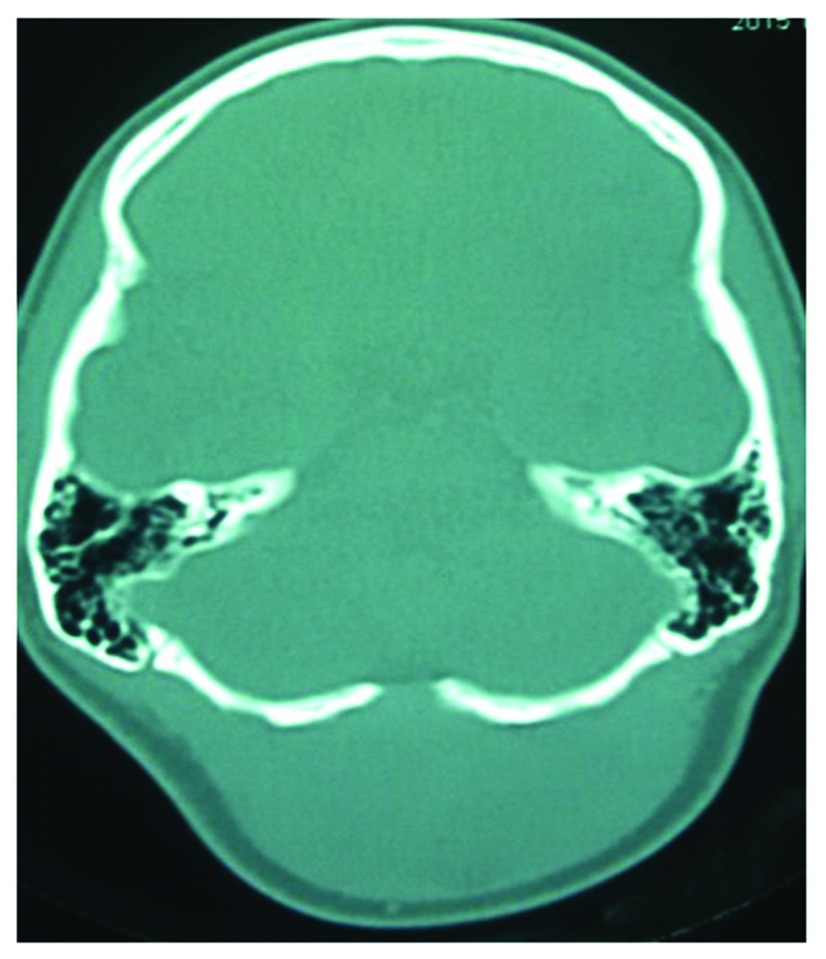
CT bone window showing a minimal osseous gap in the midline but no intracranial extension of the lesion.

A 16-year-old female from Butwal, Nepal presented to our outpatient clinic with a chief complaint of slow progressive swelling in the occipital region of the scalp, which she had been experiencing for the last 2 years. There was no history of trauma, pain, tinnitus, dizziness or discharge associated with the lesion, and no significant previous medical or surgical illnesses had been reported. Local examination revealed a soft to firm subcutaneous lesion measuring 9 × 8 cm ^2^, with no bruit within the lesion and normal overlying skin. There was no transmitted pulsation or cough impulse, and there were no palpable bony defects felt around the margins of the lesion. Lower cranial nerve examination was normal and cerebellar signs were negative. CT findings showed a homogeneously enhanced subcutaneous lesion ( Figure 1), but with no intracranial extension ( Figure 2).

**Figure 3.**
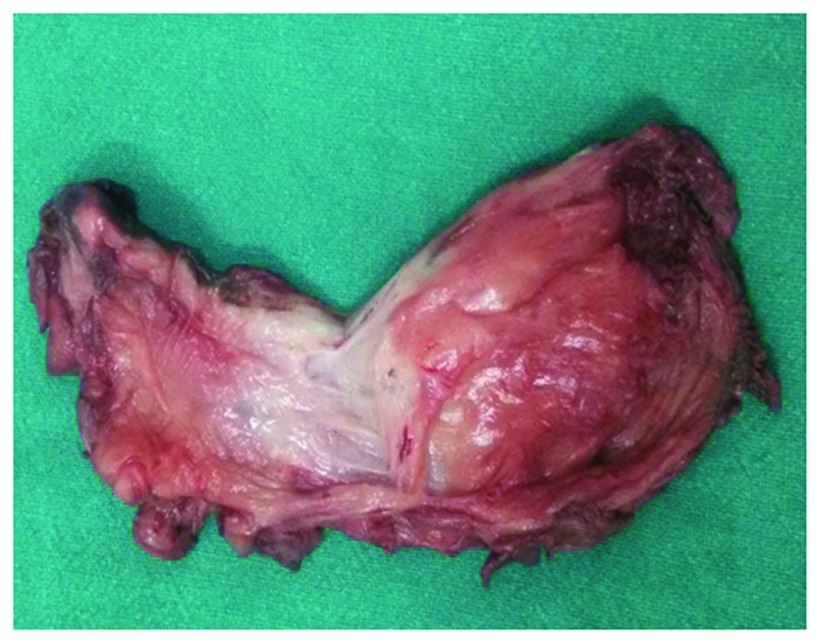
Excised specimen showing the giant lesion, of firm consistency and with no visible necrotic areas.

**Figure 4.**
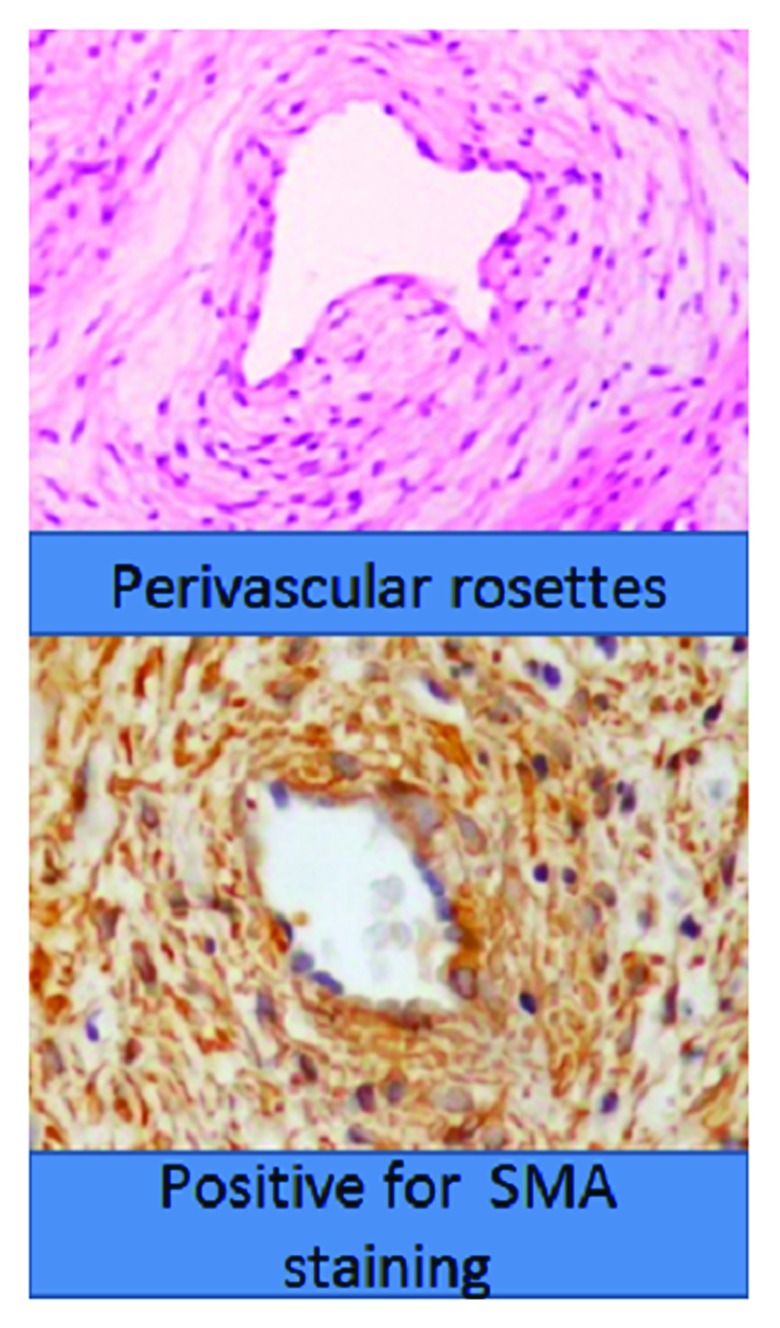
Photomicrograph of tissue taken from the lesion excised from the patient, showing characteristic spindle cells forming perivascular rosettes and staining with smooth muscle actin (SMA).

After thorough counselling and consent, the patient was booked in for excision of the lesion. Adequate blood for transfusion was supplied because of the vascularity of the scalp and the giant size of the lesion. A midline incision was given, with the patient in the prone position. The edges of the lesion were vascular, with major pedicles from bilateral occipital arteries. Complete excision was undertaken ( Figure 3). Intra-operatively, the patient was transfused two pints of blood. Post-operative recovery was uneventful land she was discharged on the third day. Histological examination of the lesion revealed presence of spindle-shaped cells, forming characteristic rosettes around the blood vessels. Positive staining for smooth muscle actin (SMA) was highly suggestive for myopericytoma ( Figure 4), and the lack of mitotic cells or tissue necrosis confirmed its benign nature. Patient follow-up took place 2 weeks later, with no symptoms and a well healed wound. She was advised to come for periodic follow-ups every month.

## Discussion

Myopericytoma has been described as being a type of perivascular tumor in the latest edition of World Health Organization classification of tumors of soft tissue and bone ^6^. Histologically it is characterized by spindle cells forming perivascular rosettes and staining positive for SMA and negative for Desmin, Bcl2 and CD34 ^9^. Though usually the size of a myopericytoma is less than 2 cm in superficial soft tissue, larger tumor size has been reported in the visceral locations ^10, 11^. Some cases of the malignant form showing invasion, mitotic figures and necrosis have been described ^6^. These malignant forms also show a high Ki-67 proliferative index, contrary to benign forms with low Ki-67 index ^12^.

Prior to diagnosing the myopericytoma, initially the major differential diagnosis was of a giant diffuse lipoma. Other differential diagnoses included other mesenchymal lesions, like desmin positive angioleiomyomas, glomus tumors in which epitheloid cells form rosettes, and solitary fibrous tumors, which do not form visible perivascular rosettes ^13^. These can be differentiated on the basis of their characteristic immunohistological reactivity patterns, such as positive staining with SMA and often also with h-Caldesmon ^9^.

Recurrence of the tumor can occur, even in benign cases, so complete excision should be the goal ^13^. Following complete excision, patients should return for periodic follow-ups despite the benign nature of the tumor.

## Conclusion

Though a rarity, myopericytoma should be ruled out prior to surgical management of subcutaneous lesions, because sometimes the high vascular nature of the lesion may impose difficulties during its excision and pose a risk to the patients’ life if adequate arrangements for blood transfusions have not been made.

## Consent

Written informed consent for publication of the patient’s details and their images was obtained from the guardian of the patient.
