# Supplementary material for: Case Report: A giant myopericytoma involving the occipital region of the scalp - a rare entity
Source: F1000Res. 2016 Dec 22;5:2905. [Version 1] doi: 10.12688/f1000research.10505.1 (PMC5302154; doi:10.12688/f1000research.10505.1)
Supplement: Peer review history

# Peer review: Referee response for version 1

**DOI:** 10.5256/f1000research.11323.r19615

**Published:** 2017-02-08

**Authors:** Umit Eroglu

**Affiliations:** 1 Department of Neurosurgery, School of Medicine, Ibni Sina Hospital, Ankara University, Ankara, Turkey

İt is a well written article and contains novel knowledge.

 It is an original article but provides minimal sufficient details for practitioners.

 It includes a background of the case's history and progression and provides details of any physical examination and diagnostic tests, treatment given and outcomes.

 It includes a discussion of the importance of the findings that also describes their relevance to future understanding of disease processes, diagnosis or treatment.

I have read this submission. I believe that I have an appropriate level of expertise to confirm that it is of an acceptable scientific standard.

# Peer review: Referee response for version 1

**DOI:** 10.5256/f1000research.11323.r19917

**Published:** 2017-02-07

**Authors:** Sanela Zukić

**Affiliations:** 1 Department of Neurology, University Clinical Center Tuzla, Tuzla, Bosnia and Herzegovina

This case is very interesting, and describes the rare entity which will serve in the medical practice.

 The title is appropriate for the content of the article and the abstract represent a suitable summary of the work. 

 The design, methods and analysis of the results from the study been explained and are they appropriate for the topic being studied.

 Also, the conclusions are sensible, balanced and justified on the basis of the results of the study.

I have read this submission. I believe that I have an appropriate level of expertise to confirm that it is of an acceptable scientific standard.

# Peer review: Referee response for version 1

**DOI:** 10.5256/f1000research.11323.r19555

**Published:** 2017-01-23

**Authors:** Ravi Dadlani

**Affiliations:** 1 Department of Neurosurgery, Apollo Speciality Hospital, Nellore, Andhra Pradesh, India

I congratulate Dr Munakomi et al for an interesting article.

 The following are my comments:

 The article is well written and highlights a rare pathological entity which may thought of in the differential diagnosis of large scalp lesions.

1. A clinical photograph outlining the lesion would be interesting.
2. There are no MRI images. Any large scalp tumor should have a pre-op MRI. If MRI images are available kindly upload those.
3. English language editing is recommended e.g. instead of using the word ‘pint’ the word ‘unit of blood’ seems more appropriate.
4. There is no discussion on the radiological characteristics of the tumor and its differential diagnosis.
5. It would be interesting to have a tabulated review of literature of myopericytomas of the scalp.
6. I would also recommend the authors provide a table of differential diagnosis of various pathologies and their immunohistochemical characterizations.
7. Although the take home message appears to be a high clinical suspicion in order to prevent excess blood loss intra-operatively, the authors do not specifically mention any particular measures, if any, they took to minimize bleeding intra-operatively.
8. A two week follow up is too short for any tumor and some discussion on how frequent (%) is the recurrence after complete excision and what is the treatment strategy for recurrences.

**Final Verdict**: Paper may be accepted for indexing with the relevant changes.

I have read this submission. I believe that I have an appropriate level of expertise to confirm that it is of an acceptable scientific standard.
